# Presentation of Caffeine Intoxication in an Active Duty Service Member Originally Believed to Have a Psychotic Disorder

**DOI:** 10.7759/cureus.18615

**Published:** 2021-10-08

**Authors:** Allison Duncan, David Dixon

**Affiliations:** 1 Surgery, F Edward Hebert School of Medicine, Uniformed Services University of the Health Sciences, Bethesda, USA; 2 Psychiatry, Brooke Army Medical Center, San Antonio, USA

**Keywords:** psychosis, military training, schizotypal personality disorder, brief psychotic disorder, caffeine intoxication

## Abstract

Caffeine has the highest use of any psychoactive substance worldwide and is highly appealing to military personnel who are required to work the long, grueling hours necessary to complete a mission. However, published reports of caffeine intoxication in the medical literature are rare.

Individuals undergoing military training experience long work hours in a stressful environment. These conditions can make various caffeine supplements enticing. However, most energy drinks contain multiple serving sizes, and supplements are often packaged in large containers, increasing individuals’ risk of caffeine overconsumption and intoxication. Additionally, young adults in stressful environments are the classic demographic in which the first presentation of a psychiatric illness, such as psychotic disorder, occurs. Interestingly, many of the presenting signs and symptoms, including agitation, restlessness, insomnia, tremors, tachycardia, and psychomotor agitation, are the same in both diagnoses. This stresses the importance of differentiating caffeine intoxication from a psychotic disorder.

We present a novel case of an active-duty Service member who presented to the emergency room with symptoms concerning the psychotic disorder. After a more detailed history was acquired, caffeine intoxication became more likely and was ultimately treated. We discuss the overlap in presentation and thus difficulty in differentiating caffeine intoxication from a psychotic disorder.

## Introduction

Caffeine has the highest use of any psychoactive substance worldwide [1]. It occurs naturally and can be found in coffee, tea, and in substantial concentrations within energy drinks and pre-workout supplements [2-5]. This is highly appealing to military personnel who are required to work the long hours necessary to complete a mission [6]. Oftentimes, energy drinks will partner with or make their military support well known to the public. Additionally, highly caffeinated beverages are easily available at multiple locations throughout military bases.

The symptoms of caffeine intoxication are known to include agitation, restlessness, insomnia, tremors, diuresis, tachycardia, gastrointestinal disturbances, and psychomotor agitation [7]. Yet, published reports of caffeine intoxication are few and far between. Given that men and women in military training are at a potentially increased risk for caffeine intoxication, considering their long work hours and potential naivety to the substance, a better understanding of its presentation would be beneficial to medical providers. Additionally, young adults in stressful environments, such as military training, are at an increased risk of experiencing their first presentation of a psychiatric illness. Thus, it is important to differentiate those disorders - especially those involving psychosis - from caffeine intoxication.

In this case report, we present a young Service member who presented to the emergency room with symptoms concerning psychotic disorder while attending Basic Officer Leaders Course (BOLC). Further discussion with the patient revealed significant caffeine ingestion and insomnia prior to presentation, supporting a diagnosis of caffeine intoxication.

## Case presentation

A 24-year-old active-duty female Service member with no formal past psychiatric history was attending the BOLC. She presented to the emergency room at the request of her command due to concern for disorganized thought processes and odd behavior.

Per her command, they received a phone call from the patient’s partner stating the patient was “having a mental breakdown,” making “threatening remarks,” talking about “a poisonous film on [her] bell peppers,” and appearing to converse with others while nobody was around her at training.

In the emergency room, the patient reported agitation, anxiety, irritability, panic, restlessness, and poor concentration. She denied suicidal or homicidal ideation and visual or auditory hallucinations at the time of presentation. Further information was difficult to gather from the patient. She repeatedly asked, “Is it okay if I give you an understanding of my framework and my timeline?” followed by a speech in a non-tangential pattern. 

Vitals in the emergency room included a blood pressure of 169/99, but her remaining vital signs were within normal limits. Her pupils were equal, round, and reactive to light with no nystagmus. The urine drug screen was negative and head computed tomography revealed no evidence of acute intracranial pathology. Complete blood count, comprehensive metabolic panel, thyroid-stimulating hormone, and urinalysis were all within normal limits.

Further questioning revealed that the patient had consumed 420 milligrams of caffeine in pre-workout prior to presentation. Additionally, she reported receiving no more than four hours of sleep per night throughout her one-month time at BOLC.

Admission to the psychiatric unit was deemed necessary, due to safety concerns, potential inability to care for herself, and so she could be monitored throughout her initial treatment. Once admitted to the unit, the patient’s behavior and conversations with hospital staff were consistently bizarre. She repeatedly screamed her partner’s name throughout hospital days 1 and 2. A nurse on the unit reported the patient speaking of a monk who helped to “keep [her] chakras in line.” And, she described a recent discovery of “sacred numbers” and “sacred patterns in geography,” then requested to be discharged at a specific time based on arbitrary calculations. 

The visual and auditory hallucinations reported prior to presentation, delusions, disorganized behavior, and disorganized speech caused brief psychotic disorder to be of great concern. Additionally, her eccentric behavior and magical thinking, both observed on the unit and described by her command, partner, and mother, all caused schizotypal personality disorder to be an underlying concern for this patient. However, the consumption of 420 milligrams of caffeine within 24 hours of presentation significantly impacted the patient’s differential and treatment plan. Caffeine intoxication became the greatest concern. 

While on the unit, her treatment primarily consisted of supportive and cognitive-based therapy. To assist with her sleep, she was prescribed nightly doses of three milligrams of Melatonin as needed for the initial four nights on the unit. When it proved to be ineffective, based on the patient’s personal reports of only receiving three to five hours of sleep per night, she consented to and then was prescribed daily doses of 25 milligrams Doxepin on hospital day 4. 

After the first dose of Doxepin, the patient reported sleeping for four hours. At that time, her mother was contacted and reported this was “normal for her.” Her safety and sleep were no longer of concern, so a psychiatric consult was made within the patient’s hometown, and she was discharged on hospital day 6.

## Discussion

It is well understood that the first presentation of a psychiatric illness often presents in a stressful environment when an individual is between their late teens and mid-20s [8]. Thus, military training is the perfect conduit for such a presentation. This patient’s demographic supports this, causing psychotic disorder to be the initial thought after she presented to the emergency room with eccentric and disorganized behavior. It was not until the team collected a more detailed history, that her high ingestion of caffeine prior to presentation was revealed and caffeine intoxication could even be considered on the differential.

Military personnel often have arduous jobs at odd hours of the day, which can be extremely energy taxing. Caffeine is not only tempting but often necessary to complete the mission in this type of working environment. It creates the perfect market for energy drinks such as Rip It, Monster, NOS, Bang, and many workout supplements that advertise between 80 and 300 milligrams of caffeine per serving [2-5]. A single serving of any of these options likely will not cause harm, as many studies have shown consumption of up to 400 milligrams of caffeine is not associated with adverse effects [9]. However, each can of an energy drink typically contains greater than one serving. Two to five servings, or greater, within a 24-hour period, can cause significant harm, including documented cases of psychosis [1,10]. The Diagnostic and Statistical Manual of Mental Disorders, Fifth Edition (DSM-5) states, “recent consumption of caffeine (typically a high dose well in excess of 250 milligrams),” as diagnostic criteria for caffeine intoxication. Additional criteria including “restlessness,” “muscle twitching,” “rambling flow of thought and speech,” and “psychomotor agitation” oftentimes overlap with those of psychosis especially when insomnia and periods of inexhaustibility lead to visual and/or auditory hallucinations [7]. This stresses the importance of differentiating between the two diagnoses - caffeine intoxication and psychotic disorder. However, with so much overlap in the symptoms and little objective information necessary for diagnosis, this can cause a challenge to providers. Cases discussing how the two diagnoses have been differentiated in the past could not be found in the literature, causing us to believe this situation is a rarity. In our limited experience, documented in this case, a detailed history ultimately allowed us to narrow down the differential.

It may be beneficial to further study the similarities and differences in the presentations of caffeine intoxication and brief psychosis to better differentiate the two. Additionally, it would be advantageous to look further into the effects of high caffeine consumption in young adults within a stressful environment.

## Conclusions

Young individuals who join the military often are exposed to high-intensity working conditions with increased stress, potentially increasing their risk of caffeine intoxication. Their naivety to the substance and long working hours which require high energy levels cause them to be more vulnerable to the toxicities of the caffeine contained within energy drinks and workout supplements. Thus, it is crucial that medical providers are able to recognize the signs and symptoms of caffeine intoxication when it presents. However, the similarities between caffeine intoxication and psychotic disorder can make this a challenge. This stresses the importance of collecting a thorough history each time an individual presents with signs and symptoms concerning psychotic disorder and keeping caffeine intoxication on the differential when the psychotic disorder is being considered.
